# Dietary *Echinacea angustifolia* Extract Enhances Antioxidant and Immune Responses and Disease Resistance in Common Carp, *Cyprinus carpio*

**DOI:** 10.1155/anu/9410394

**Published:** 2026-11-01

**Authors:** Morteza Yousefi, Hamed Abdollahpour, Hossein Adineh, Sadaf Seyfollahi, Ali Taheri Mirghaed, Seyyed Morteza Hoseini

**Affiliations:** ^1^ Department of Veterinary Medicine RUDN University 6 Miklukho-Maklaya Street Moscow 117198 Russia rudn.ru; ^2^ Comparative Endocrinology and Integrative Biology Center of Marine Sciences University of Algarve Faro Portugal ualg.pt; ^3^ Department of Fisheries Faculty of Agriculture and Natural Resources Gonbad Kavous University Gonbad Kavous Golestan Iran gonbad.ac.ir; ^4^ Department of Aquatic Animal Health Faculty of Veterinary Medicine University of Tehran Tehran Iran ut.ac.ir; ^5^ Inland Waters Aquatics Resources Research Center Iranian Fisheries Sciences Research Institute Agricultural Research, Education and Extension Organization Gorgan Iran ifsri.ir

**Keywords:** *Aeromonas hydrophila*, *Echinacea angustifolia*, growth performance, mucosal immunity, phytogenic feed additive

## Abstract

This study evaluated the effects of dietary supplementation with a hydroalcoholic extract of *Echinacea angustifolia* (EE) on growth performance, feed utilization, mucosal and systemic immunity, antioxidant status, and disease resistance in common carp, *Cyprinus carpio*, fingerlings. Fish (initial body weight: 4.88 ± 0.08 g) were fed four experimental diets containing 0 (control), 1%, 2%, and 4% EE for 8 weeks. Growth performance, intestinal digestive enzyme activities, skin mucus and intestinal immune parameters, hepatic, plasma, and kidney antioxidant indices, stress indicators (cortisol and glucose), and post‐challenge mortality against *Aeromonas hydrophila* were evaluated. The EE extract exhibited a 2,2‐diphenyl‐1‐picrylhydrazyl (DPPH) radical‐scavenging activity of 95.5% ± 0.31% and an IC50 of 10.7% against *A. hydrophila* in vitro. The results demonstrated that dietary EE supplementation led to significant quadratic responses in growth performance and feed efficiency. Fish fed the 1% and 2% EE diets showed significantly higher final weight (10.00–10.13 g), weight gain (104.4%–106.8%), daily growth rate (DGR), specific growth rate (SGR), and production efficiency (PE) than those fed the 4% EE diet (9.30 g; 90.0%), whereas these variables did not differ significantly from the control (9.62 g; 99.7%). 1% and 2% EE significantly decreased feed conversion ratio (FCR; 1.43–1.45), compared with the control group (1.74) and the lowest FCR was observed in the 2% EE group. All EE‐supplemented groups exhibited significantly enhanced skin mucus immune parameters (increased lysozyme, total immunoglobulin (Ig), hemolytic activity, protease, peroxidase, ALP, and bactericidal activities) as well as intestinal immune and antioxidant parameters (elevated lysozyme, hemolytic activity, superoxide dismutase [SOD], and glutathione peroxidase [GPx], along with reduced malondialdehyde [MDA] levels). Following the *A. hydrophila* challenge, EE‐fed fish showed significantly lower mortality (20.0%–28.9% vs. 56.7% in control), attenuated cortisol and glucose stress responses, and better‐preserved antioxidant status across the liver, plasma, and kidney. Correlation analysis revealed three distinct clusters (growth–immunity, stress–immunity, and antioxidant–disease resistance), while exploratory random forest analysis identified mucosal immune parameters (mucus Ig, mucus bactericidal activity, and mucus lysozyme) as the variables most strongly associated with mortality, explaining 78.4% of the variance. The 4% inclusion level provided no additional benefit over the 1%–2% diets for most parameters. Overall, dietary EE did not improve growth relative to the control but enhanced feed efficiency, mucosal and systemic immunity, antioxidant defense, and disease resistance, with 1%–2% inclusion producing the most favorable responses among the tested levels under the present experimental conditions.

## 1. Introduction

Aquaculture is one of the fastest‐growing food production sectors globally, providing a crucial source of animal protein for human consumption [1]. Among freshwater aquaculture species, common carp, *Cyprinus carpio*, is one of the most widely cultivated finfish worldwide, with global production exceeding 4 million tons annually [1]. However, intensive aquaculture practices have led to increased susceptibility to infectious diseases, particularly bacterial pathogens, such as *Aeromonas hydrophila*, which cause substantial economic losses and threaten the sustainability of carp farming [2, 3]. *A. hydrophila* is a Gram‐negative, opportunistic pathogen responsible for motile aeromonad septicemia, characterized by hemorrhagic lesions and systemic organ failure, often resulting in high mortality rates in intensive culture systems [3].

Traditionally, antibiotics and chemotherapeutics have been employed to prevent and control disease outbreaks in aquaculture. However, the overuse and misuse of antibiotics have raised severe concerns regarding antimicrobial resistance, environmental pollution, and tissue residues, posing significant hazards to both environmental integrity and public health [3]. Furthermore, the emergence of multidrug‐resistant strains of *A. hydrophila* has prompted regulatory restrictions on prophylactic antibiotic usage globally, necessitating the development of sustainable, eco‐friendly disease management strategies [4]. Consequently, functional feed additives derived from medicinal plants (phytogenics) have gained considerable interest as viable antibiotic alternatives due to their immunostimulatory, antioxidant, antimicrobial, and growth‐promoting properties [5]. For example, dietary phytogenic compounds improved fish resistance against *A. hydrophila* infection by modulating innate immunity [6, 7].

Phytochemicals, including phenolic compounds, flavonoids, polysaccharides, and alkaloids, enhance innate immune responses, alleviate oxidative stress, and improve disease resistance in various fish species [8, 9]. Among phytogenic candidates, the genus *Echinacea*, native to North America, has a well‐established history of use for its medicinal properties [10]. The primary bioactive constituents of *Echinacea* spp. include alkamides, caffeic acid derivatives (such as echinacoside, cichoric acid, and caftaric acid), polysaccharides, and glycoproteins, which act synergistically to modulate physiological and immune functions [10]. Alkamides act as cannabinoid receptor type 2 (CB2) agonists to modulate cytokine synthesis, while caffeic acid derivatives confer potent radical‐scavenging activity, and polysaccharides (e.g., arabinogalactan) stimulate macrophage activity and pro‐inflammatory signaling [11].

The immunomodulatory and protective effects of *Echinacea* spp. have been documented in several teleosts. Dietary supplementation with *Echinacea* spp. extracts (mainly *Echinacea purpurea*) improved growth, nonspecific immunity, antioxidant defenses, and disease resistance in rainbow trout, *Oncorhynchus mykiss* [12], Nile tilapia, *Oreochromis niloticus* [13], common carp [14], grass carp, *Ctenopharyngodon idella* [15], and flathead gray mullet, *Mugil cephalus* [16]. *Echinacea* spp. can increase serum lysozyme and complement activities, stimulated leukocyte respiratory burst, upregulation of antioxidant enzymes, and suppressed lipid peroxidation [17, 18], alongside direct in vitro and in vivo antagonism against fish pathogens [14, 19].

Despite these documented benefits, existing aquaculture research has overwhelmingly focused on *E. purpurea*, leaving the potential of *Echinacea angustifolia* comparatively unexplored. *E. angustifolia* possesses a distinct phytochemical profile, characterized by substantially higher concentrations of lipophilic alkamides, particularly dodeca‐2E,4E‐dienoic acid isobutylamide, which acts as a CB2 receptor agonist and modulates cytokine synthesis and immune cell function [10, 20]. Additionally, *E. angustifolia* contains higher concentrations of echinacoside, a caffeic acid derivative with documented antioxidant and antibacterial activities, compared to *E. purpurea* [10, 11]. These compositional differences suggest that *E. angustifolia* may exert distinct immunomodulatory effects and potentially different physiological responses in fish, warranting direct investigation rather than assuming equivalence with *E. purpurea*. Furthermore, systemic immune responses to *Echinacea* spp. have been documented, mucosal immunity, particularly the skin mucus layer, which represents the primary physical and biochemical barrier against waterborne pathogen entry [21], remains largely unaddressed. Phytogenic additives have been shown to enhance skin mucus immune parameters, such as lysozyme, complement activity, ALP, total Ig, and bactericidal activity in fish [22–24], yet comprehensive multitissue assessments evaluating the systemic antioxidant network (hepatic, renal, and plasma compartments) in tandem with intestinal health and predictive modeling are also lacking.

Therefore, the present study was conducted to evaluate the effects of dietary *E. angustifolia* hydroalcoholic extract (EE; 0, 1%, 2%, and 4%) on growth performance, digestive enzyme activities, mucosal and systemic immunity, multitissue antioxidant capacity, physiological stress responses, and post‐challenge resistance against *A. hydrophila* in common carp fingerlings. Pearson correlation and exploratory random forest analyses were used to identify associations among physiological parameters and to rank variables by their relative importance in explaining mortality, with the understanding that these approaches are hypothesis‐generating and do not establish causality.

Based on the existing literature and the bioactive profile of E. angustifolia, we hypothesized that (1) dietary EE would enhance mucosal and systemic immune responses and antioxidant defense in a dose‐dependent manner, with 1%–2% inclusion producing the most favorable responses among the tested levels; (2) EE supplementation would reduce post‐challenge mortality against *A. hydrophila* and attenuate the associated endocrine stress responses; and (3) intestinal and mucosal immune/antioxidant parameters would be the strongest physiological variables associated with disease resistance, as assessed by correlation and exploratory random forest analyses.

## 2. Materials and Methods

### 2.1. Ethical Consideration

This study was conducted after approval of the Scientific Board of the Faculty of Fisheries, University of Gonbad Kavous, Iran (241; 657003. Date: 2025/10/7).

### 2.2. EE Preparation and Analysis

EE was purchased from Giahkala Co. (Mashhad, Iran). It was a hydroalcoholic (70% ethanol) extract of the root and aerial parts of *E. angustifolia* at 1:1 ratio.

Individual phenolic compounds were determined by LC–MS/MS following previously published procedures [25]. Analyses were conducted using an API 4000 QTRAP hybrid triple‐quadrupole/linear ion‐trap mass spectrometer (Applied Biosystems, Darmstadt, Germany) equipped with an electrospray ionization (ESI) source. Chromatographic separation was achieved on a Gemini C18 column (50 mm × 2 mm; 5 μm particle size; 110 Å pore size; fully porous organo‐silica).

Each sample injection (10 μL) was analyzed over 5.5 min at a column temperature of 40°C. The mass spectrometer was operated in positive ESI mode using selected‐ion monitoring (SIM) with a monitoring window of 0.70 amu. The nebulizer pressure was set at 55 psi, drying‐gas flow was 1 mL/min, and skimmer voltage ranged from 20 to 80 V. Positive‐ion detection provided improved sensitivity for the relatively acidic *E. angustifolia* extract. Data were collected and processed using Workstation Method Builder software.

To assess the bactericidal activity of EE, *Aeromonas hydrophila* (PTCC 1890) was cultured on nutrient agar at 30°C for 48 h. The bacterial cells were suspended in physiological saline at a concentration of 10^8^ colony‐forming units (CFU)/mL. A 400 μL aliquot of the suspension was mixed with 100 μL of EE at concentrations of 1%, 2%, 4%, 8%, and 16% and incubated at room temperature for 1 h. Following incubation, the mixtures were serially diluted (10^−1^–10^−7^) and plated on nutrient agar. After 48 h of incubation at 30°C, the number of CFU was counted. The bactericidal effect was calculated as the percentage inhibition relative to the untreated control. Finally, the concentration required to achieve 50% inhibition was determined by plotting the percentage of bacterial growth inhibition against the EE concentrations.

Antioxidant capacity of EE was determined by 2,2‐diphenyl‐1‐picrylhydrazyl (DPPH) method [26]. Briefly, a 0.1 mM methanolic solution of DPPH was prepared in the dark. Subsequently, 300 µL of the extract was mixed with 700 µL of the DPPH solution, and the resulting mixture was incubated in the dark at room temperature for 30 min. The absorbance of the sample and the control solution (containing all reagents except EE) was measured at 517 nm using a spectrophotometer. The percentage of DPPH radical scavenging activity was calculated using the following equation:

Scavenging activity %=Acontrol−AsampleAcontrol×100,

where *A*_control_ was the absorbance of the control (DPPH solution without the sample) and *A*_sample_ was the absorbance of the DPPH solution mixed with the sample extract.

### 2.3. Diets

A basal diet was formulated based on the nutritional requirements of juvenile common carp (Table 1). Four experimental diets were prepared including zero or control levels (0EE), 1% (1EE), 2% (2EE), and 4% (4EE) of EE (equivalent to 0, 10, 20, and 40 g/kg diet). To prepare the diets, the dry feed ingredients were first thoroughly homogenized in an electric mixer for 20 min. Then, desired amounts of EE were added to water to a final total volume of 400 mL, which were mixed with 1000 g feed ingredient mixture, making into a paste. The resulting dough was kneaded for 15 min and pelleted using a meat grinder with a die of 3 mm pore diameter. The pellets were dried at room temperature/shade and stored at 4°C until consumption. The control diet received the same volume of water (400 mL per 1000 g feed mixture) and underwent the same preparation and drying process as the EE‐supplemented diets, with only the EE omitted. The chemical composition and proximate analysis of the diets including crude protein (Kjeldahl method), crude fat (Soxhlet/ether extraction method), crude ash (incineration in a 550°C oven), crude fiber (acid–base digestion), and moisture (drying in an oven at 105°C) were measured according to standard guidelines [27].

**Table 1:** Ingredients and chemical composition of the basal diet.

Ingredients	Dietary EE levels (%)			
	0	1	2	4
Meat meal^a^	334	334	334	334
Soybean meal	230	230	230	230
Wheat meal	400	400	400	400
Sunflower oil	7	7	7	7
Canola oil	7	7	7	7
L‐lysine^b^	7	7	7	7
L‐methionine^b^	5	5	5	5
Vitamin premix^c^	5	5	5	5
Mineral premix^d^	5	5	5	5
Proximate composition				
Dry matter	892.2	890.0	896.9	894.7
Crude protein	360.8	359.8	361.2	361.4
Crude lipid	59.4	60.0	58.9	59.1
Ash	62.7	65.7	63.9	67.1
Energy (MJ/kg)	17.2	17.3	17.3	17.4

*Note:* Data are presented as g/kg.

^a^Makianmehr Co., Golestan, Iran.

^b^Morghenojan.Co., Tehran, Iran.

^c^Vitamin premix (per kg of diet): vitamin A, 2000 IU; vitamin B_1_ (thiamin), 5 mg; vitamin B_2_ (riboflavin), 5 mg; vitamin B_6_, 5 mg; vitamin B_12_, 0.025 mg; vitamin D_3_, 1200 IU; vitamin E, 63 mg; vitamin K_3_, 2.5 mg; folic acid, 1.3 mg; biotin, 0.05 mg; pantothenic acid calcium, 20 mg; inositol, 60 mg; ascorbic acid (35%), 110 mg; niacinamide, 25 mg.

^d^Mineral premix (per kg of diet): MnSO_4_, 10 mg; MgSO_4_, 10 mg; KCl, 95 mg; NaCl, 165 mg; ZnSO_4_, 20 mg; KI, 1 mg; CuSO_4_, 12.5 mg; FeSO_4_, 105 mg; Co, 1.5 mg.

### 2.4. Fish Husbandry and Growth Performance

Common carp fingerlings (unsexed) were obtained from a local breeding and rearing facility and transported to the laboratory. The fish were kept in a holding tank (1500 L) for 10 days to acclimatize to the laboratory conditions and the control diet, offered at 3% of biomass divided into three meal a day. After the acclimatization period, 360 fish, visually healthy, with an initial average weight of 4.88 ± 0.08 g, were randomly distributed to 12 laboratory tanks (including four dietary treatments and three replicates for each treatment; 30 fish in each tank), each containing 60 L of dechlorinated tap water. The fish and tanks were randomly allocated following simple randomization. The feeding period lasted for 8 weeks and the fish were fed 3% of the biomass, divided into three meals (no feed wastes were observed in the treatments). In order to adjust the feeding rate, fish biomasses were measured every 2 weeks. The tanks were continuously aerated and about 30% of the water in each tank was siphoned daily and replaced with clean water. Water quality parameters including temperature (24.0 ± 0.65°C), dissolved oxygen (6.3 ± 0.62 mg/L), pH (7.85 ± 0.36), and total ammonia (0.19 ± 0.03 mg/L) were measured weekly. At the end of the rearing period, daily growth rate (DGR), specific growth rate (SGR), feed conversion ratio (FCR), production efficiency (PE), and survival were calculated as follow:

DGR g/day=Wf−Wit; SGR %/day=100100×lnWf−lnWit; FCR=FIWf−Wi; PE g/L=Bf60; Survival %=×NfNi,

where *W*_i_ is the initial weight (g), *W*_f_ is the final weight (g), *t* is the rearing period (56 days), FI is the amount of feed consumed (g), *B*_f_ is the final biomass per tank (g), *N*_*i*_ is the initial number of fish, and *N*_*f*_ is the final number of fish at the end of the period.

### 2.5. Bacterial Challenge

At the end of the 8‐week feeding period, fish from different groups were exposed to the pathogenic bacteria *A. hydrophila* PTCC 1890. The bacterium was cultured on nutrient agar at 30°C for 48 h. Then, the cells were harvested and suspended in sterile 0.85% NaCl solution to reach an optical density of 0.09 at 600 nm (0.5 McFarland). To reach a cell density of 1 × 10^7^ CFU/mL, the stock suspension was serially diluted in NaCl solution. The final suspension was intraperitoneally injected into the fish at 100 μL/fish (approximately equal to 10^5^ CFU/g live weight) [28]. Fifteen fish per tank were randomly selected and injected by the bacterial suspension. The fish were returned to their corresponding tank and post‐challenge mortality was monitored for 14 days.

### 2.6. Sampling

Sampling was performed randomly (simple randomization). Sampling was performed in two stages, including the end of the feeding period (pre‐challenge; skin mucus, intestine, liver, kidney, and plasma were sampled) and 12 h after the pathogenic challenge test (post‐challenge; liver, kidney, and plasma were sampled) from three fish per replicate (nine fish per treatment). Fish were anesthetized using eugenol (100 mg/L) before the sampling. The replicate was a fish tank in the present study; hence, the sub‐samples per replicate were pooled to form a single sample per tank, at each sampling time.

The skin mucus was collected indirectly by placing a fish in a polyethylene bag (15 cm × 15 cm) containing 5 mL physiological saline. After a gentle rubbing, the mixture was extracted into a microtube and centrifuged at 13,200 × *g* for 20 min at 4°C and the supernatant was stored at −70°C for analysis [29]. Blood was collected from the caudal peduncle using heparin‐soaked syringes. Plasma was separated by blood centrifugation (5000 × *g* for 7 min at 4°C) and frozen at −70°C until assay. After blood collection, the fish were decapitated, and the liver, kidney, and intestine were quickly removed. The tissues were homogenized in cold phosphate buffer, and after centrifugation (13,200 × *g* for 20 min at 4°C), the supernatant was separated for enzymatic and oxidative assays and frozen at −70°C.

### 2.7. Biochemical Analysis

Biochemical analyses were conducted following simple randomization to minimize the potential confounders derived from the order of treatments and measurements and fish/tank location. All analyses were conducted blindly by labeling the samples with confidential codes.

### 2.8. Intestinal Digestive Enzyme Activity

The enzyme activity was determined using the specific substrate of each enzyme and spectrophotometric methods. The amount of soluble protein in the enzyme extracts was also determined by the pyrogallol‐red method and using a commercial biochemical kit (Zist Chem Co., Tehran, Iran) to express the enzyme activity in terms of activity units per mg of soluble protein. Amylase activity was determined using alpha‐starch as the substrate. After incubation of the enzyme extract with the starch solution, the amount of reducing sugars produced was measured by adding the 3,5‐dinitrosalicylic acid (DNS) reagent. The absorbance of the samples after heating and cooling was read at the appropriate wavelength, and the enzyme activity was calculated based on the amount of maltose released [30].

Protease activity was assessed using the AZO‐casein method [31]. After incubation of the intestinal extract with the substrate, the reaction was stopped by the addition of trichloroacetic acid. The absorbance of the supernatant, which indicated the amount of peptide compounds or tyrosine released, was then read spectrophotometrically. One unit of protease activity was defined as the amount of enzyme that produced one microgram of tyrosine per min under the test conditions.

Lipase activity was measured using a specific lipid substrate, p‐nitrophenyl myristate [32]. The release of p‐nitrophenol upon hydrolysis of the substrate by lipase was determined by measuring the optical absorbance. The enzyme activity was expressed as the amount of p‐nitrophenol produced per unit time and relative to the soluble protein of the intestinal extract.

### 2.9. Nonspecific Immune Indices of the Skin Mucus

#### 2.9.1. Lysozyme

The lysozyme activity of the skin mucus was determined based on the turbidimetric method using a suspension of the bacteria *Micrococcus luteus*. For this purpose, 900 μL bacterial suspension (prepared in phosphate buffer) was mixed with 100 μL of the skin mucus extract. The decrease in turbidity of the suspension due to the destruction of the bacterial cell wall by lysozyme was recorded at a wavelength of 500 nm for 5 min. One unit of lysozyme activity was defined as a decrease of 0.001 units of optical absorption per minute. The results were reported in units per mg of protein after adjustment based on the protein content of the sample solution [33].

#### 2.9.2. Total Immunoglobulin (Ig)

Total Ig of skin mucus was measured by the polyethylene glycol (PEG) precipitation method according to the method of Siwicki and Anderson [34]. First, the total protein concentration of the mucus sample was determined. Then, equal volumes of the mucus sample and 12% PEG solution were mixed together and kept in cold conditions for 2 h to precipitate the Igs. After centrifugation of the samples, the protein concentration in the supernatant was determined. The difference between the total protein before precipitation and the remaining protein after precipitation was considered as the amount of total Ig.

#### 2.9.3. Hemolytic Activity

Skin mucus hemolytic activity, as an indicator of the function of the complement system, was measured using sheep red blood cells. First, the sheep red blood cells were washed with barbital buffer containing EGTA, magnesium, and gelatin to provide the necessary conditions for the evaluation of the alternative complement pathway. After appropriate dilution (0.313%–10%), the mucus samples were mixed with the red blood cell suspension and incubated for 90 min at room temperature. The hemolysis reaction was stopped by adding buffer containing EDTA and the samples were centrifuged. The absorbance of the supernatant was measured at 412 nm, and the degree of hemolysis was calculated relative to the complete hemolysis of red blood cells in distilled water [35].

#### 2.9.4. Peroxidase Activity

Skin mucus peroxidase activity was measured based on the oxidation of the substrate 3,3^′^,5,5^′^‐tetramethylbenzidine hydrochloride (TMB) in the presence of hydrogen peroxide. In this method, the mucosal sample was mixed with a solution of TMB and hydrogen peroxide and incubated for a specified time. The enzymatic reaction was stopped by adding sulfuric acid and the intensity of the color formed was read using a spectrophotometer or microplate reader at the appropriate wavelength. Peroxidase activity was expressed using the absorbance of the sample and after adjustment based on the protein content of the mucus extract [36].

#### 2.9.5. Bactericidal Activity

The bactericidal activity of mucocutaneous against *A. hydrophila* PTCC 1890 was determined by the colony count method. For this purpose, 100 μL of mucus extract was mixed with an equal volume of the suspension of *A. hydrophila* bacteria (McFarland scale: 0.5) and incubated at room temperature. After 1 h, serial dilutions of the resulting mixture were prepared and cultured on nutrient agar plates at 30°C for 48 h. After incubation, the plates were counted for the number of colonies formed. The percentage of bactericidal activity was calculated by comparing the number of CFU in the sample containing mucus with the control group without mucus:

Bactericidal activity %=1 −CFU sample/CFU control×100.

### 2.10. Gut Immune and Antioxidant Parameters

Lysozyme activity and hemolytic activity in gut extract were measured by the *M. luteus* turbidimetric method and the sheep red blood cell hemolysis method, respectively, as described for skin mucus. In all tissue assays, the values were standardized based on the soluble protein concentration of the tissue extract.

#### 2.10.1. Superoxide Dismutase (SOD)

SOD activity was determined based on the enzyme’s ability to inhibit the autooxidation of pyrogallol [37]. In this assay, pyrogallol spontaneously oxidizes in an alkaline medium, producing an increase in light absorption; the presence of SOD slows down this process. The changes in optical absorption of the samples were recorded at the appropriate wavelength, and the enzyme activity was calculated based on the percentage of inhibition of pyrogallol autoxidation. One SOD unit was defined as the amount of enzyme that could inhibit 50% of pyrogallol autoxidation. The results were expressed in units of activity per milligram of soluble protein.

#### 2.10.2. Catalase (CAT)

CAT activity was measured based on the decomposition of hydrogen peroxide [38]. The tissue extract was mixed with a hydrogen peroxide solution and incubated at the appropriate temperature. After the end of the reaction time, the remaining hydrogen peroxide was measured by the formation of a yellow complex by adding ammonium molybdate. The optical absorption of the samples was read at a wavelength of 405 nm and the CAT activity was calculated using a standard curve or the relevant formula. The results were reported as CAT activity units per mg of soluble protein.

#### 2.10.3. Glutathione Peroxidase (GPx)

GPx activity was determined based on the ability of this enzyme to consume reduced glutathione (GSH) to neutralize peroxides [39]. In this method, tissue extracts were incubated in the presence of GSH and hydrogene peroxide. The amount of GSH remaining after the reaction was measured using the 5,5^′^‐dithiobis‐(2‐nitrobenzoic acid) (DTNB) reagent. The product of the reaction of DTNB with sulfhydryl groups was read at 412 nm. GPx activity was expressed as the amount of GSH consumed per unit time and relative to the amount of soluble protein.

#### 2.10.4. Total Antioxidant Capacity (TAC)

TAC was determined by the iron reducing power (FRAP) method. The basis of this method is the reduction of ferric ion (Fe^3+^) to ferrous ion (Fe^2+^) by antioxidant compounds present in the sample. The resulting ferrous ion reacts with an appropriate reagent and forms a colored complex, the intensity of which is proportional to the antioxidant capacity of the sample. The optical absorption at 700 nm was recorded and the results were calculated using a standard curve [40].

#### 2.10.5. Malondialdehyde (MDA)

The concentration of MDA as an indicator of lipid peroxidation was measured by the thiobarbituric acid (TBA) reaction method [41]. In this method, MDA present in the tissue extract reacts with TBA under acidic and thermal conditions and forms a colored complex. After cooling and separation of interfering components, the absorbance of the colored solution was recorded with a spectrophotometer at a wavelength of 532 nm. The amount of MDA was calculated using the molar absorption coefficient or standard curve and expressed relative to the soluble protein of the sample.

### 2.11. Antioxidant Parameters of Liver and Kidney

SOD, CAT, and GPx activities as well as TAC and MDA levels in the liver and kidney were measured according to the methods described for intestine samples. GSH levels were also determined by the Ellman reagent method [42]. The sample was mixed with a trichloroacetic acid solution and after centrifugation, the supernatant was reacted with DTNB reagent. DTNB is converted to a yellow compound, 5‐thio‐2‐nitrobenzoic acid, in the presence of free thiol groups of GSH. The optical absorbance of the samples was read at 412 nm, and the GSH concentration was calculated using a standard curve.

### 2.12. Plasma Stress and Immune Indices

#### 2.12.1. Cortisol

Plasma cortisol concentration was measured by ELISA using a commercial kit from Monobind Co. (Ohio, USA). The assay was performed according to the manufacturer’s instructions, and the optical absorbance of the reactions was read at 450 nm with a microplate reader. The cortisol concentration of the samples was calculated using a standard curve prepared from the kit standards. The measurement range of the kit used was 0–500 ng/mL, and its intra‐assay and inter‐assay coefficients of variation were 5.48% and 5.67%, respectively.

#### 2.12.2. Glucose

Plasma glucose concentration was measured using a commercial kit from Zist Chem Co. (Tehran, Iran) based on the glucose‐oxidase method. In this method, glucose in the sample is oxidized by the enzyme glucose oxidase to produce hydrogen peroxide. Hydrogen peroxide then reacts with a chromogen in the presence of peroxidase to form a colored compound. The resulting color intensity, which is proportional to the sample glucose concentration, was read at the wavelength specified by the kit with a spectrophotometer or a microplate reader. The glucose concentration was calculated by comparing the optical absorbance of the samples and the kit standard and reported in mg/dL.

#### 2.12.3. Ascorbate

The amount of ascorbate in plasma was determined by manual colorimetric method [43]. In this method, plasma proteins were first removed using a precipitating agent to prevent protein interference. Then, ascorbate in the supernatant reduced the color reagent in an oxidation–reduction reaction, and the resulting color change was measured spectrophotometrically. The ascorbate concentration of the samples was calculated based on a standard curve prepared from ascorbic acid and reported in appropriate units.

### 2.13. Plasma Lysozyme, Natural Hemolytic Activity, and Total Ig

Plasma lysozyme activity was measured by the turbidimetric method using a suspension of *M. luteus*, as described for skin mucosa. Plasma natural hemolytic activity was also determined by measuring the percentage of hemolysis of sheep red blood cells, as described above for skin mucus. The total Ig content of plasma was also determined by selective precipitation of Igs with 12% PEG, as described above for skin mucus.

### 2.14. Statistical Analysis

For parameters measured at the end of the 8‐week feeding trial (e.g., growth performance, feed utilization, and survival), data were first checked for the normality and homogeneity of variances. Percentage data were arcsine square‐root transformed prior to the analysis. Data were subjected to one‐way analysis of variance (ANOVA), and when significant differences were detected, pairwise comparisons among dietary treatments were performed using Tukey’s honestly significant difference (HSD) test. In addition, linear and quadratic orthogonal polynomial contrasts were evaluated to determine response trends to dietary EE levels. Because dietary levels (0, 1%, 2%, and 4%), were unequally spaced, contrast coefficients were specifically derived for unequal intervals following [44] to ensure mutual orthogonality. For physiological, stress, and immune parameters measured before and after the challenge, data were analyzed using repeated‐measures ANOVA with the tank as the subject (experimental unit) and time (pre‐ vs. post‐challenge) as the within‐subject factor. Mauchly’s test was used to verify sphericity. When the main effect of dietary EE or interaction effect of time × dietary EE was significant, pairwise comparisons were conducted using Tukey’s HSD test. Mortality percentages were arcsine square‐root transformed prior to one‐way ANOVA, followed by the Tukey’s HSD test for pairwise comparisons. In all cases, *α* was set to be 0.050. The statistical software, SPSS v.22, was used for the analysis.

Pearson correlation analysis was performed to evaluate relationships among growth, immunity, stress, antioxidant, and mortality parameters using tank‐level data (*n* = 12). A comprehensive correlation matrix was constructed using the cor function in R with complete‐case analysis. The following variables were included: growth parameters (SGR, FCR, and weight gain), mucus immune parameters (lysozyme, Ig, hemolytic activity, and bactericidal activity), gut immune and antioxidant parameters (lysozyme, CH50, SOD, GPx, MDA, and CAT), plasma immune and stress parameters (lysozyme, ACH50, Ig, cortisol, glucose, TAC, and ascorbate), and mortality. Correlation heatmaps were generated using the corrplot package with a blue–white–red color scheme (blue = negative correlation and red = positive correlation) and hierarchical clustering to visualize three distinct clusters: (1) growth–immunity, (2) stress–immunity, and (3) antioxidant–disease resistance. Correlation coefficients (*r*) and *p*‐values were calculated for each pairwise comparison, and *p*‐values were adjusted for multiple testing using the Benjamini–Hochberg false discovery rate (FDR) procedure.

Exploratory random forest regression was performed to identify physiological variables associated with post‐challenge mortality using tank‐level data (*n* = 12). All measured immune, antioxidant, and stress parameters (*n* = 23 variables) were initially considered as candidate predictors, without pre‐selection based on correlation with mortality, to avoid circularity and data dredging. A random seed was set (set.seed = 123) to ensure reproducibility. The random forest model was implemented using the randomForest package in R with 500 trees and default settings for the number of variables tried at each split (mtry = p/3 for regression). The out‐of‐bag (OOB) error estimate was calculated to provide an internal validation and an unbiased estimate of model performance. Variable importance was assessed using the percentage increase in mean squared error (%IncMSE), where higher values indicate greater importance for predicting mortality. The proportion of variance explained was calculated as 1 − (MSE / Var(y)). Random forest analysis is presented as an exploratory, hypothesis‐generating approach, and the identified variables should be interpreted as associations rather than causal determinants, particularly given the limited sample size (*n* = 12). Variable importance was visualized as a horizontal bar plot with purple gradient coloring (light purple = low importance and dark purple = high importance) to maintain consistency with the PCA figures.

## 3. Results

### 3.1. Characteristics of EE

DPPH scavenging capacity of EE was 95.5% ± 0.31%, whereas, IC50 for bactericidal activity was 10.7% EE concentration. Main phenolic compounds of EE are listed in Table 2, showing that caffeic acid (119 mg/mL), luteolin (68.1 mg/mL), p‐coumaric acid (36.6 mg/mL), rutin (36.2 mg/mL), and gallic acid (22.6 mg/mL) were the most abundant compounds in EE.

**Table 2:** Results of LC‐MS analysis of phenolic/flavonoid compounds.

Detected compounds	Concentration (mg/mL)
Caffeic acid	119
Luteolin	68.1
p‐Coumaric acid	36.6
Rutin	36.2
Gallic acid	22.6
Apigenin	18.4
Myricetin	15.5
p‐OH‐Benzoic acid	11.6
Ferulic acid	5.8
Kaempferol	5.5
Chlorogenic acid	5.4
Fumaric acid	5.1
Quercetin	4.9

### 3.2. Growth Performance and Feed Utilization

Growth performance, feed efficiency, and survival of common carp fingerlings after the 8‐week feeding trial are summarized in Table 3. Dietary EE levels significantly affected FBW, WG, DGR, SGR, and PE (*p* < 0.05). These parameters exhibited significant quadratic responses to dietary EE levels (*p* < 0.05), although linear trends were also significant for these metrics. Specifically, fish fed 1% and 2% EE diets achieved significantly higher FBW, WG, DGR, SGR, and PE compared to the 4% EE group, while the control group showed intermediate values that did not differ significantly from the 1% and 2% EE treatments.

**Table 3:** Growth performance, feed efficiency, and survival of common carp fingerlings (*n* = 3) after 8 weeks feeding on EE‐supplemented diets.

Growth/production parameters	Dietary EE levels (%)				Pooled SEM	*p*‐Values		
	0	1	2	4		ANOVA	Linear	Quadratic
Initial weight (g)	4.82	4.84	4.96	4.90	0.02	0.058	0.077	0.077
Final weight (g)	9.62^ab^	10.00^b^	10.13^b^	9.30^a^	0.11	0.004	0.037	0.001
Weight gain (%)	99.74^ab^	106.76^b^	104.41^b^	90.04^a^	2.23	0.009	0.010	0.008
DGR (%)	1.78^ab^	1.91^b^	1.86^b^	1.61^a^	0.04	0.009	0.010	0.008
SGR (%/d)	1.24^ab^	1.30^b^	1.28^b^	1.15^a^	0.02	0.008	0.009	0.008
PE (g/L)	4.81^ab^	5.00^b^	5.07^b^	4.65^a^	0.06	0.004	0.037	0.001
FCR	1.74^b^	1.45^a^	1.43^a^	1.71^b^	0.05	0.006	0.619	0.001
Survival (%)	100	100	100	100	0.00	1.000	1.000	1.000

*Note:* Different superscript lowercase letters within a row show significant differences among the treatments (Tukey HSD test).

Abbreviations: DGR, daily growth rate; FCR, feed conversion ratio; PE, production efficiency; SGR, specific growth rate.

Furthermore, FCR was significantly affected by dietary treatments (*p* = 0.006) and displayed a significant quadratic trend (*p* = 0.001), without a significant linear trend (*p* = 0.619). The lowest FCR was observed in the 2% EE group, which was significantly improved compared to the control and 4% EE groups. Survival was 100% across all dietary treatments with no significant differences (*p* = 1.000).

### 3.3. Intestinal Digestive Enzymes’ Activity

The activities of intestinal digestive enzymes are shown in Table 4. Dietary EE levels significantly influenced gut amylase activity (*p* = 0.042), displaying a significant quadratic trend (*p* = 0.026) without a significant linear response (*p* = 0.090). Amylase activity was significantly elevated in the 2% EE group compared to the control (*p* < 0.05), whereas fish fed 1% and 4% EE diets exhibited intermediate activities that did not differ significantly from the control or 2% EE groups. In contrast, gut lipase and protease activities were not significantly affected by dietary EE supplementation (*p* > 0.05) and showed neither significant linear nor quadratic responses (*p* > 0.05).

**Table 4:** Intestinal digestive enzymes’ activity of common carp fingerlings (*n* = 3) after 8 weeks feeding on EE‐supplemented diets.

Digestive enzymes	Dietary EE levels (%)				Pooled SEM	*p*‐Values		
	0	1	2	4		ANOVA	Linear	Quadratic
Amylase (U/mg protein)	1.37^a^	1.60^ab^	2.10^b^	1.73^ab^	0.10	0.042	0.090	0.026
Lipase (U/mg protein)	13.33	16.00	19.33	18.33	0.97	0.105	0.051	0.125
Protease (U/mg protein)	33.00	32.33	30.33	35.67	1.03	0.366	0.353	0.171

*Note:* Different superscript lowercase letters within a row show significant differences among the treatments (Tukey HSD test).

### 3.4. Skin Mucus Immunological Parameters

Skin mucus immunological parameters are presented in Table 5. Dietary EE levels significantly affected all evaluated mucosal immune parameters, including lysozyme, Total Ig, hemolytic activity, protease, peroxidase, ALP, and bactericidal activity (*p* < 0.05). Furthermore, all of these parameters exhibited both significant linear (*p* < 0.05) and quadratic responses (*p* < 0.05) to dietary EE supplementation.

**Table 5:** Skin mucus immunological parameters of common carp fingerlings (*n* = 3) after 8 weeks feeding on EE‐supplemented diets.

Immunological parameters	Dietary EE levels (%)				Pooled SEM	*p*‐Values		
	0	1	2	4		ANOVA	Linear	Quadratic
Lysozyme (U/mg protein)	17.67^a^	28.67^b^	28.00^b^	30.33^b^	1.59	<0.001	0.000	0.003
Total Ig (mg/mL)	14.33^a^	18.33^ab^	19.67^b^	19.33^b^	0.78	0.025	0.015	0.033
Hemolytic activity (%)	24.33^a^	33.00^b^	33.00^b^	32.00^b^	1.22	0.003	0.009	0.003
Protease (U/mg protein)	32.00^a^	42.67^b^	42.67^b^	42.33^b^	1.51	0.002	0.003	0.003
Peroxidase (U/mg protein)	2.27^a^	3.10^b^	3.30^b^	3.23^b^	0.15	0.013	0.010	0.017
ALP (U/mg protein)	1.50^a^	2.40^b^	2.53^b^	2.60^b^	0.15	0.004	0.002	0.012
Bactericidal activity (%)	22.33^a^	51.00^b^	53.33^b^	53.67^b^	4.58	0.008	0.007	0.014

*Note:* Different superscript lowercase letters within a row show significant differences among the treatments (Tukey HSD test).

Abbreviations: ALP, alkaline phosphatase; Ig, immunoglobulin.

Post hoc pairwise comparisons revealed that all EE‐supplemented diets (1%, 2%, and 4% EE) resulted in significantly higher mucus lysozyme, hemolytic, protease, peroxidase, ALP, and bactericidal activities compared to the control group (*p* < 0.05), with no significant differences observed among the three EE‐supplemented groups. In addition, skin mucus total Ig was significantly elevated only in the 2% and 4% EE groups compared to the control (*p* < 0.05), while the 1% EE group displayed intermediate levels that did not differ significantly from the control or the higher EE inclusion levels.

### 3.5. Intestinal Immunological and Antioxidant Parameters

Intestinal immunological and antioxidant parameters are shown in Table 6. Dietary EE levels significantly influenced all evaluated intestinal immune and antioxidant parameters (*p* < 0.05). Moreover, all measured parameters exhibited both significant linear (*p* < 0.05) and quadratic (*p* < 0.05) responses to dietary EE supplementation.

**Table 6:** Intestinal immunological and antioxidant parameters of common carp fingerlings (*n* = 3) after 8 weeks feeding on EE‐supplemented diets.

Immunological/antioxidant parameters	Dietary EE levels (%)				Pooled SEM	*p*‐Values		
	0	1	2	4		ANOVA	Linear	Quadratic
Lysozyme (U/mg protein)	53.33^a^	76.00^b^	83.00^b^	83.33^b^	4.19	0.006	0.003	0.015
Hemolytic activity (%)	13.00^a^	29.67^b^	33.33^b^	32.67^b^	2.76	0.002	0.002	0.005
SOD (U/mg protein)	1.53^a^	2.33^b^	3.47^c^	2.70^bc^	0.22	<0.001	0.001	0.000
CAT (U/mg protein)	1.87^b^	1.20^a^	1.33^ab^	1.27^a^	0.10	0.026	0.036	0.043
GPx (U/mg protein)	1.57^a^	3.27^b^	2.83^b^	2.73^b^	0.21	0.001	0.017	0.001
MDA (mM/g WW)	11.30^b^	6.43^a^	6.03^a^	6.00^a^	0.73	0.001	0.001	0.002

*Note:* Different superscript lowercase letters within a row show significant differences among the treatments (Tukey HSD test).

Abbreviations: CAT, catalase; GPx, glutathione peroxidase; MDA, malondialdehyde; SO, superoxide dismutase.

Post hoc pairwise comparisons revealed that all EE‐supplemented diets (1%, 2%, and 4% EE) significantly enhanced intestinal lysozyme, hemolytic, and GPx activities, while significantly reducing MDA levels compared to the control group (*p* < 0.05). Intestinal CAT activity was significantly decreased in fish fed the 1% and 4% EE diets compared to the control (*p* < 0.05), whereas the 2% EE group exhibited intermediate activity. Intestinal SOD activity was significantly elevated in all EE‐supplemented groups compared to the control, with the highest activity recorded in the 2% EE group, which was significantly higher than that in the 1% EE group (*p* < 0.05).

### 3.6. Disease Resistance

Post‐challenge cumulative mortality was significantly influenced by dietary EE levels (*p* < 0.05). The mortality rate followed significant linear and quadratic trends in response to dietary EE supplementation (*p* < 0.05). All EE‐supplemented groups (1%, 2%, and 4% EE) exhibited significantly lower mortality compared to the control group (*p* < 0.05). While no significant differences were observed among the EE‐supplemented treatments, the 2% EE group displayed the numerically lowest mortality (Figure 1).

**Figure 1:**
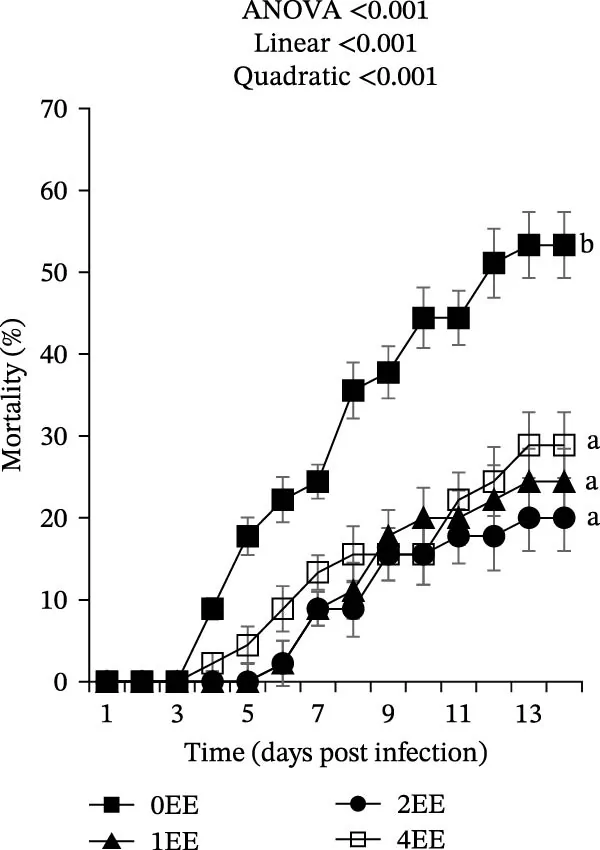
Mortality of common carp fingerlings (*n* = 3) after 8 weeks feeding on EE‐supplemented diets and experimentally infection with *A. hydrophila*. Different lowercase letters in front the markers show significant differences among the treatments, 14 days after infection (Tukey HSD test).

### 3.7. Hepatic Antioxidant Status

The effects of dietary EE on hepatic antioxidant parameters are illustrated in Figure 2. Hepatic SOD and CAT activities significantly increased after the challenge. The dietary EE levels significantly affected post‐challenge SOD levels, with the 2EE and 4EE groups exhibiting higher activities than the 0EE group, while the 1EE group did not differ from the other groups. Conversely, CAT activity was not significantly affected by dietary EE levels.

**Figure 2:**
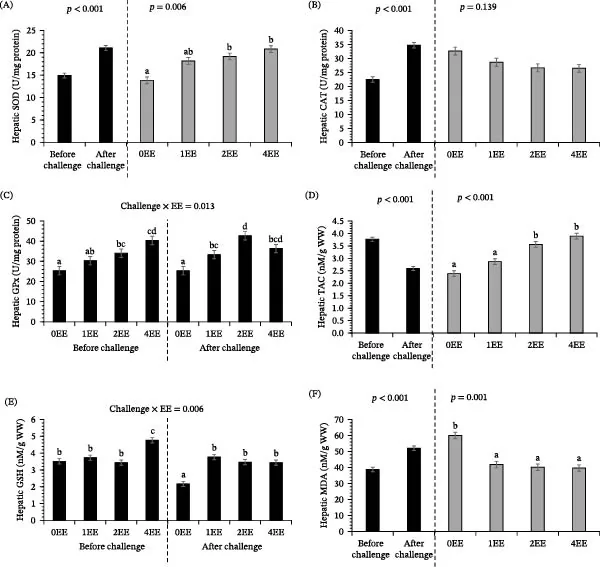
Hepatic antioxidant status. (A) SOD, (B) CAT, (C) GPx, (D) TAC, (E) GSH, and (F) MDA levels in fish fed different levels of EE (0EE, 1EE, 2EE, and 4EE) before and after the challenge. Different lowercase letters above the bars show significant differences among dietary EE groups (main effects) or dietary EE × time (interaction effects) according to Tukey HSD test.

A significant interaction between the challenge and dietary treatments was observed for hepatic GPx activity. The 2EE and 4EE showed significantly higher hepatic GPx activity compared to the 0EE group before the challenge. All EE‐treated groups had significantly higher hepatic GPx compared to the 0EE group after the challenge; however, only the 2EE group showed significant increase compared to the corresponding pre‐challenge level.

Hepatic TAC levels decreased after the challenge, whereas MDA levels increased. Post‐challenge, dietary EE supplementation significantly affected both parameters; the 2EE and 4EE groups maintained higher TAC levels and lower MDA concentrations than the 0EE group.

A significant interaction between the challenge and dietary treatments was observed for the hepatic GSH content. Hepatic GSH levels significantly increased in the 4EE group compared to the 0EE group before the challenge. All EE‐treated fish had significantly higher hepatic GSH levels compared to the 0EE group after the challenge. Hepatic GSH levels decreased in the 0EE and 4EE treatments after the challenge compared to the corresponding levels before the challenge.

### 3.8. Plasma and Kidney Antioxidant Indices

The responses of plasma and kidney antioxidant indices to the challenge and dietary treatments are presented in Figure 3. Plasma TAC levels did not differ between the pre‐ and post‐challenge sampling times. However, dietary EE administration significantly affected plasma TAC, with all EE‐supplemented groups (1EE, 2EE, and 4EE) exhibiting higher values than the 0EE group. Plasma ascorbate levels decreased after the challenge. Furthermore, fish fed EE‐supplemented diets exhibited higher plasma ascorbate levels than the 0EE group, while no significant differences were observed among the EE‐supplemented groups.

**Figure 3:**
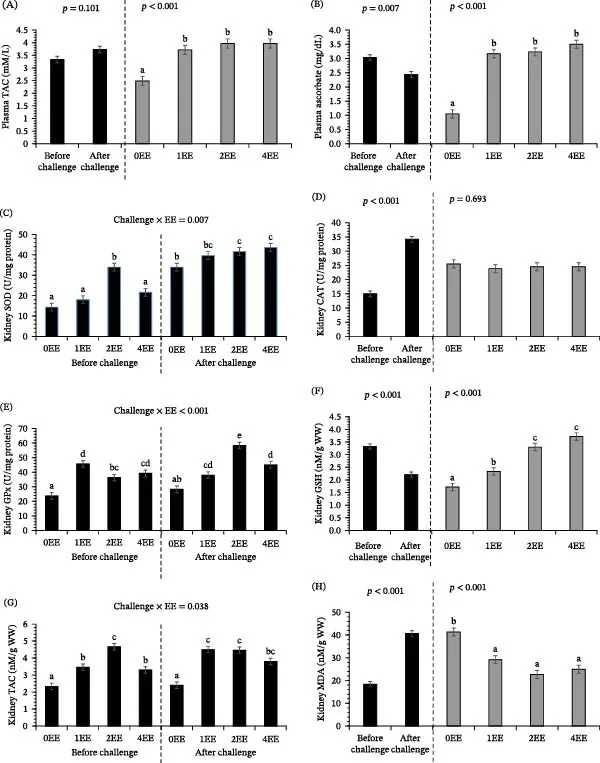
Plasma and kidney antioxidant indices. (A) Plasma TAC, (B) plasma ascorbate, (C) kidney SOD, (D) kidney CAT, (E) kidney GPx, (F) kidney GSH, (G) kidney TAC, and (H) kidney MDA levels in fish fed different levels of EE (0EE, 1EE, 2EE, and 4EE) before and after the challenge. Different lowercase letters above the bars show significant differences among dietary EE groups (main effects) or dietary EE × time (interaction effects) according to Tukey HSD test.

There were interaction effects of dietary EE and challenge on the kidney SOD, GPx, and TAC. The kidney SOD activity significantly increased in the 2EE treatment before the challenge compared to the 0EE group. There was no significant difference in the kidney SOD activity among the treatments at this time. Bacterial challenge significantly increased the kidney SOD in all treatments, and the 2EE and 4EE had significantly higher SOD compared to the 0EE group.

Kidney CAT activity increased after the challenge, but it was not significantly affected by the dietary treatments.

All EE‐treated groups showed significantly higher kidney GPx compared to the 0EE group, both before and after the challenge. The highest GPx activity was observed in the 1EE and 2EE groups before and after the challenge, respectively. Furthermore, the enzyme’s activity increased in the 2EE group after the challenge, when compared to the corresponding level before the challenge.

Kidney GSH levels decreased after the challenge. Dietary treatment also affected kidney GSH, with a stepwise increase from 0EE to 4EE, such that the 4EE group showed the highest value, followed by 2EE, 1EE, and 0EE.

All EE‐treated groups showed significantly higher kidney TAC compared to the 0EE group, both before and after the challenge. The highest TAC activity was observed in the 2EE and 1EE/2EE groups before and after the challenge, respectively. Moreover, TAC increased in the 1EE group after the challenge, when compared to the corresponding level before the challenge.

Kidney MDA levels increased after the challenge. Dietary treatment significantly affected post‐challenge kidney MDA levels, where all EE‐supplemented groups (1EE, 2EE, and 4EE) exhibited lower MDA levels than the 0EE group, with no significant differences among the EE‐supplemented groups.

### 3.9. Plasma Stress Indicators

Plasma stress indicators are presented in Figure 4. A significant interaction between the challenge and dietary treatments was observed for plasma cortisol levels. There was no significant difference in plasma cortisol levels before the challenge among the treatments. While all treatments showed significant cortisol elevation after the challenge, the EE‐treated groups had significantly lower cortisol levels than that of the 0EE group.

**Figure 4:**
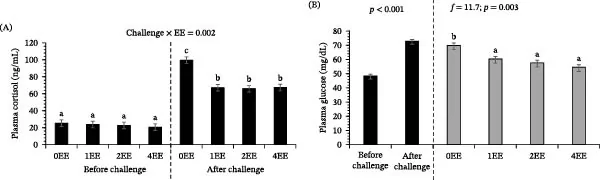
Plasma stress indicators. (A) Cortisol and (B) glucose levels in fish fed different levels of EE (0EE, 1EE, 2EE, and 4EE) before and after the challenge. Different lowercase letters above the bars show significant differences among dietary EE groups (main effects) or dietary EE × time (interaction effects) according to Tukey HSD test.

Plasma glucose levels significantly increased after the challenge. The dietary EE levels significantly affected post‐challenge glucose levels, where the 1EE, 2EE, and 4EE groups showed lower glucose levels than the 0EE group. No significant differences were observed among the EE‐supplemented groups.

### 3.10. Plasma and Kidney Immune Indices

The immune responses in the plasma and kidney are shown in Figure 5. A significant interaction between the challenge and dietary treatments was observed for plasma lysozyme activity. Plasma lysozyme significantly increased in 2EE and 4EE groups, compared to the 0EE group, before the challenge. Plasma lysozyme decreased after the challenge in the 0EE group compared to corresponding level before the challenge. All EE0treated groups had significantly higher plasma lysozyme activity, compared to the 0EE group, after the challenge.

**Figure 5:**
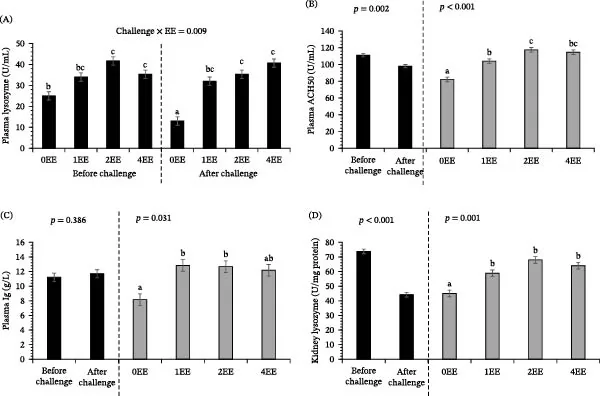
Plasma and kidney immune indices. (A) Plasma lysozyme, (B) plasma ACH50, (C) plasma Ig, and (D) kidney lysozyme levels in fish fed different levels of EE (0EE, 1EE, 2EE, and 4EE) before and after the challenge. Different lowercase letters above the bars show significant differences among dietary EE groups (main effects) or dietary EE × time (interaction effects) according to Tukey HSD test.

Plasma ACH50 activity decreased after the challenge. Dietary treatment significantly increased plasma ACH50 activities in all EE‐supplemented groups (1EE, 2EE, and 4EE) compared to the 0EE group. The highest ACH50 activity was related to the 2EE treatment.

Plasma Ig levels did not differ between the pre‐ and post‐challenge sampling times. However, dietary treatment significantly affected plasma Ig levels, where the 1EE and 2EE groups showed higher Ig levels than the 0EE group. No significant differences were observed in plasma I levels among the EE‐supplemented groups.

Kidney lysozyme activity decreased after the challenge. Dietary treatment significantly affected post‐challenge kidney lysozyme activity, with the 1EE, 2EE, and 4EE groups exhibiting higher activity than the 0EE group, and no significant differences were observed among the EE‐supplemented groups.

### 3.11. Correlation Analysis Results

Pearson correlation analysis was performed to evaluate relationships among growth, immunity, stress, antioxidant, and mortality parameters (Figure 6). The comprehensive correlation heatmap revealed three distinct clusters of significant correlations. Growth–Immunity Cluster: SGR showed weak correlations with immune parameters (*r* = −0.05 to 0.13), while FCR demonstrated moderate negative correlations with mucus lysozyme (*r* = −0.39) and mucus Ig (*r* = −0.43), indicating that better feed efficiency is associated with enhanced immune status. Weight gain showed weak correlations with gut immunity parameters (*r* = −0.01 to −0.10). Stress–immunity cluster: Plasma cortisol levels showed moderate to strong negative correlations with immune parameters, including mucus bactericidal activity (*r* = −0.65), mucus lysozyme (*r* = −0.56), and plasma lysozyme (*r* = −0.34). Plasma glucose was negatively correlated with gut lysozyme (*r* = −0.64) and positively correlated with mortality (*r* = 0.43). Mortality showed strong negative correlations with mucus lysozyme (*r* = −0.87), gut lysozyme (*r* = −0.73), and plasma TAC (*r* = −0.78). Antioxidant–disease resistance cluster: gut GPx showed the strongest negative correlation with mortality (*r* = −0.87), followed by gut SOD (*r* = −0.75) and plasma ascorbate (*r* = −0.88). Gut MDA, a marker of oxidative damage, showed the strongest positive correlation with mortality (*r* = 0.92), confirming that reduced oxidative stress is strongly associated with improved disease resistance. Plasma TAC was negatively correlated with mortality (*r* = −0.78).

**Figure 6:**
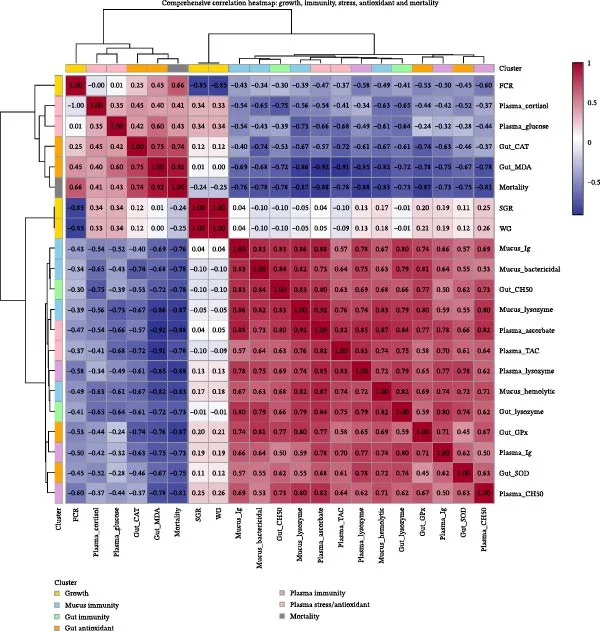
Comprehensive correlation heatmap showing relationships among growth parameters (SGR, FCR, and WG), immune parameters (mucus: lysozyme, Ig, hemolytic, and bactericidal; gut: lysozyme and CH50; plasma: lysozyme, CH50, and Ig), stress indicators (cortisol and glucose), antioxidant parameters (SOD, GPx, MDA, CAT, TAC, and ascorbate), and mortality. Red indicates positive correlation and blue indicates negative correlation. Color intensity represents correlation strength. Three distinct clusters are visible: (1) growth‐immunity (FCR shows moderate negative correlations with immune parameters, *r* = −0.39 to −0.43; SGR and WG show weak correlations), (2) stress‐immunity (cortisol and glucose show moderate to strong negative correlations with immunity, *r* = −0.34 to −0.65; glucose and cortisol show positive correlations with mortality, *r* = 0.43), and (3) antioxidant‐disease (GPx, SOD, ascorbate, and TAC show strong negative correlations with mortality, *r* = −0.75 to −0.88; MDA shows the strongest positive correlation with mortality, *r* = 0.92).  ^∗^ *p* < 0.05;  ^∗∗^ *p* < 0.010. The three clusters highlight distinct biological pathways through which EE supplementation improves health outcomes in common carp.

### 3.12. Random Forest Analysis

The OOB *R* ^2^ was 0.78, indicating that the model explained 78.4% of the variance in mortality. The OOB mean squared error was 38.53. Variable importance analysis revealed that mucus Ig was the variable most strongly associated with mortality (%IncMSE = 4.26), followed by mucus bactericidal activity (%IncMSE = 4.21), mucus lysozyme (%IncMSE = 4.06), gut GPx (%IncMSE = 3.95), and plasma ascorbate (%IncMSE = 3.87) (Figure 7). These results suggest that mucosal immune parameters, particularly Ig and bactericidal activity, may be the variables most strongly associated with disease resistance in common carp. However, given the limited sample size (*n* = 12) and the exploratory nature of this analysis, these findings should be interpreted as hypothesis‐generating rather than confirmatory.

**Figure 7:**
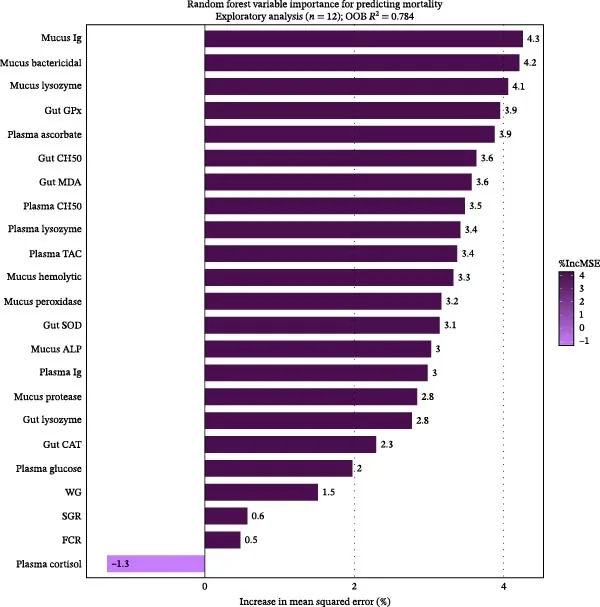
Exploratory random forest variable importance plot showing the physiological variables most strongly associated with mortality in common carp fed EE‐supplemented diets. All 23 measured physiological parameters were initially included in the model, without pre‐selection based on correlation with mortality, to avoid circularity and data dredging. The model was run on tank‐level data (*n* = 12). The out‐of‐bag (OOB) *R* ^2^ was 0.78, indicating that the model explained 78.4% of the variance in mortality. Variable importance is measured as the percentage increase in mean squared error (%IncMSE) when each variable is permuted. The purple color gradient (light → dark purple) indicates increasing importance. Mucus Ig was the variable most strongly associated with mortality, followed by mucus bactericidal activity and mucus lysozyme. Variables with negative %IncMSE (e.g., plasma cortisol) indicate that their inclusion did not improve the model’s predictive performance. Given the limited sample size (*n* = 12), these results should be interpreted as exploratory and hypothesis‐generating rather than confirmatory.

## 4. Discussion

### 4.1. Growth Performance, Digestive Enzymes, and Intestinal Health

The results indicated that EE had positive effects on feed efficiency, particularly at 1%–2% inclusion levels. Although there is no study assessing the efficacy of EE on growth performance, other *Echinacea* spp. have been reported to increase the growth rate and feed efficiency in Nile tilapia, *Oreochromis niloticus* [13], flathead gray mullet, *Mugil cephalus* [16], rainbow trout, *Oncorhynchus mykiss* [12], and guppy, *Poecilia reticulata* [45]. Differences in plant species and extracted parts explain the differences in the outcomes of the present study compared to the previous ones.

The fish were fed a fixed ration, so the EE‐induced improvement in the FCR might be related to improved nutrient utilization, partitioning, and digestive efficiency rather than simply stimulating feed intake. This may be supported by the elevated intestinal amylase activity in EE‐fed fish, potentially facilitating efficient carbohydrate digestion and assimilation. Similarly, the highest gut amylase activity, growth, and feed efficiency were observed at 2% dietary medicinal herb level in *Megalobrama amblycephala*, whereas the highest trypsin and lipase activities were observed at 1%–2% and 1% levels, respectively [46]. Furthermore, the highest growth and feed efficiency were accompanied by an increase in gut digestive enzymes in *Siniperca chuatsi* fed mulberry leaf extract [47]. This might increase non‐protein energy resources, rendering more dietary protein for growth. The underlying mechanism of action likely involves improved digestive system functions [48, 49].

The intestinal data further confirm the protective roles of EE at the primary site of digestion and immune surveillance. Elevated intestinal lysozyme, hemolytic activity, SOD, and GPx, combined with reduced MDA, show that EE enhanced gut immunocompetence and redox homeostasis while mitigating lipid peroxidation. Interestingly, the concomitant decrease in intestinal CAT activity, alongside elevated SOD and GPx and reduced MDA, further reflects a superior intestinal redox state rather than compromised protection. While GPx possesses a high substrate affinity (low *K*_*m*_) for hydrogen peroxide (H_2_O_2_) and efficiently neutralizes it at basal or physiological concentrations, CAT has a relatively lower affinity (high *K*_*m*_) and is primarily mobilized during severe, pathological H_2_O_2_ overload [50]. Thus, the subdued CAT activity indicates that steady‐state H_2_O_2_ levels were kept sufficiently low by efficient GPx clearance, preventing lipid peroxidation without triggering compensatory CAT upregulation. Excessive reactive oxygen species impair epithelial barrier competence, digestive capacity, and antioxidant enzyme activities [51, 52]. There is no study on the effects of *Echinacea* spp. on fish gut health; however, *E. purpurea* polysaccharides similarly reduced MDA and elevated TAC, SOD, and GPx across the serum, liver, and intestine in poultry [53]. This potent antioxidant protection is attributed to the high phenolic and flavonoid content of EE (e.g., caffeic acid, chlorogenic acid, quercetin, and rutin), which exert radical‐scavenging and metal‐chelating activities [54–58].

### 4.2. Pre‐Challenge Intestinal and Skin Mucus Antioxidant and Immune Parameters

One of the major known benefits of herbal feed additives is improving disease resistance [59]. Bacterial challenge with *A. hydrophila* demonstrated that EE improved disease resistance, with lower mortality in EE‐fed groups. Such a survival elevation is similar to the findings in previous studies in Guppy [45] and common carp [14], in challenge with *Aeromonas bestiarum* and *A. hydrophila*, respectively. Phytochemicals, such as phenolics and alkylamides, disrupt bacterial cell membranes and inhibit pathogen proliferation [60], providing a multifaceted defense when combined with immune and antioxidant stimulation. Supporting this, EE showed antagonistic properties against *A. hydrophila* under in vitro conditions in the present study.

The enhancement of skin mucus immunity is a major finding of this study, as the mucus layer serves as the primary physical and chemical barrier in teleosts [21]. Pre‐challenge elevations in mucus lysozyme, total Ig, ALP, protease, peroxidase, hemolytic, and bactericidal activities demonstrate robust mucosal defense priming. While *Echinacea* spp. has been widely investigated for systemic immunity [12, 14, 45], mucosal endpoints have been largely overlooked, making this one of the first reports evaluating dietary EE on common carp skin mucus. These mucosal immunostimulatory patterns parallel findings from other phytogenics, which enhanced mucus lysozyme, complement, ALP, total Ig, and bactericidal activity [22–24].

Exploratory random forest analysis identified mucosal immune parameters (mucus Ig, mucus bactericidal activity, and mucus lysozyme) as the variables most strongly associated with mortality, explaining 78.4% of variance in post‐challenge survival. While intraperitoneal injection primarily triggers systemic pathogenesis bypassing the external skin barrier, experimental evidence shows that infected teleosts can shed virulent bacteria into the surrounding water via feces, excretions, and cutaneous micro‐lesions [61]. This increase water bacterial load to infectious levels; then, skin mucus immunity is the core immune defense to protect the fish against the second infection. Supporting this, it has been shown that shedding rate of Aeromonas‐infected fish was around 10^5^–10^7^ CFU/fish/h [62], which is clearly high enough to infect the fish. Hence, the present results suggest that mucosal immune parameters, rather than intestinal antioxidant status alone, may be the variables most strongly associated with disease resistance in common carp. Several complementary mechanisms may explain these associations.

### 4.3. Pre‐ and Post‐Challenge Antioxidant Responses

In the present study, EE demonstrated strong in vitro radical‐scavenging ability, a property likely attributable to its phenolic content [63]. However, the radical‐scavenging properties of *Echinacea* spp. remain under‐documented in teleosts, research in crucian carp has shown that *E. purpurea* elevates antioxidant enzyme activities (SOD, CAT, and GR) while reducing serum hydroxyl radicals and MDA levels [17]. Furthermore, that study demonstrated that *Echinacea*‐derived polyphenols can modulate antioxidant‐related miRNAs (e.g., miR‐21, miR‐155, miR‐125b, and miR‐223), which in turn regulate the expression of SOD1, SOD2, CAT, and HO‐1. It is plausible that similar underlying transcriptional mechanisms contributed to the antioxidant outcomes observed in the present study; however, this hypothesis warrants further investigation and validation.

The superior disease resistance in EE‐fed fish was strongly linked to the robust preservation of antioxidant defenses across the liver, plasma, and kidney. Following the challenge, EE‐fed fish maintained significantly higher activities of key antioxidant enzymes (SOD, CAT, and GPx) and nonenzymatic antioxidants (GSH, TAC, and ascorbate), alongside lower MDA levels compared to the control group. This preservation of the antioxidant network is critical, as these enzymes serve as the primary defense barrier against the oxidative stress, severe inflammation, and apoptosis that typically characterize bacterial infection [55, 64]. By preventing enzymatic depletion and lipid peroxidation, the EE treatment maintained tissue homeostasis, thereby enabling the fish to better withstand the bacterial challenge.

Because the liver governs metabolic detoxification, while plasma and kidney reflect systemic redox status and excretory function, this coordinated response indicates that EE acted systemically rather than exclusively within the gut. Such multitissues studies are rare in fish, but the results are partially in line with previous studies reporting improvements in antioxidant parameters in spleen, serum, and liver of fish after dietary administration of herbal extracts [65–68]. Furthermore, random forest analysis identified plasma TAC as a reliable predictor of survival, confirming that systemic radical‐scavenging capacity played a key role in mitigating infection‐induced tissue damage. Supporting this, it has been suggested that proper antioxidant capacity aid higher immune responses and disease resistance in fish [69].

### 4.4. Pre‐ and Post‐Challenge Immune Responses

The negative correlations between FCR and immune parameters indicate an association between improved feed efficiency and enhanced immune competence in EE‐fed common carp. Fish with lower FCR values tended to exhibit stronger mucosal and systemic immune responses, suggesting that improved nutrient utilization may support the energetic and metabolic demands of immune surveillance, tissue repair, and stress adaptation (refer to [70]). Rather than representing independent responses, growth efficiency and immune competence may therefore be interconnected outcomes of improved intestinal function and overall physiological condition. Similarly, Nile tilapia showed higher resistance to *A. hydrophila* infection along with higher growth rate when fed *E. purpurea*‐supplemented diets [13]. This improved physiological status was reflected in the disease‐challenge results. Although survival did not differ significantly among the 1%, 2%, and 4% EE groups, all EE‐fed fish showed enhanced resistance relative to the control group. Survival following bacterial exposure is a multifactorial endpoint determined not only by pathogen burden, but also by the ability of the host to mount rapid innate immune responses, preserve tissue integrity, and limit oxidative and inflammatory damage [69, 71]. Therefore, the absence of significant differences among EE concentrations may indicate that the tested concentrations all provided sufficient immunological protection. The observed protection is consistent with previous studies demonstrating the disease‐preventive potential of dietary *Echinacea* spp. in teleosts. Beneficial effects have been reported against *A. bestiarum* in guppy [45]; *Vibrio anguillarum* in rainbow trout [72]; *Photobacterium damselae* in gray mullet [16]; and *A. hydrophila* in common carp [14]. Collectively, these studies support the view that *Echinacea* supplementation improves host preparedness before infection and strengthens the capacity to withstand pathogen exposure.

Prechallenge immune priming may be particularly important in explaining these protective effects. Although cellular immune variables were not evaluated in the present study, previous investigations have shown that *Echinacea* spp. supplementation can enhance phagocytic activity and leukocyte respiratory burst in rainbow trout and gray mullet [16, 72]. Such a response was also observed in rainbow trout fed a diet supplemented with *E. angustifolia* essential oil [18]. These responses are central components of early innate immunity because they facilitate pathogen recognition, engulfment, and intracellular killing. Thus, enhanced cellular activity may have contributed to the improved resistance of EE‐fed fish by allowing a faster and more effective initial response to bacterial invasion.

Humoral immune activation provides an additional explanation for the improved challenge outcome. Lysozyme contributes directly to bacterial cell‐wall degradation [73], whereas the complement system promotes opsonization, inflammatory‐cell recruitment, and pathogen lysis [74]. However, *A. hydrophila* infection can impair these humoral defenses through different mechanisms [75, 76], as shown in fish [77, 78]. Consistent with this pattern, lysozyme and complement activities decreased after bacterial challenge in the present study. Nevertheless, the EE‐treated groups maintained significantly higher activities of both parameters than the control group, indicating that EE supplementation partially mitigated the infection‐associated suppression of humoral immunity. The preservation of lysozyme and complement activity may have enhanced bacterial clearance and contributed to the higher disease resistance observed in EE‐fed fish. The mechanisms by which EE prevented or attenuated the post‐challenge decline in these parameters remain unclear and require further investigation. Nevertheless, previous studies have reported increased serum lysozyme and complement activities in rainbow trout fed a diet supplemented with *E. angustifolia* essential oil [18], as well as grass carp and rainbow trout fed *Echinacea* spp.‐supplemented diets [15, 19].

The immunostimulatory effects of *Echinacea* are likely mediated by its diverse bioactive compounds, including alkylamides, cichoric acid, and heteropolysaccharides. These compounds have been associated with macrophage activation and cytokine regulation, including stimulation of IL‐2‐related immune signaling [79–81]. Moreover, evidence from hybrid snakehead suggests that *E. purpurea* can modulate immune‐related miRNAs, including miR‐125, miR‐138, miR‐181, and miR‐10. These miRNAs are involved in the regulation of inflammatory cytokines, Igs, and antimicrobial peptides in both systemic and mucosal immune compartments [82]. Although these molecular responses were not measured here, they provide a reasonable mechanistic basis for the coordinated enhancement of immune function and disease resistance observed in EE‐fed fish. However, further direct analyses are necessary to confirm this concept.

### 4.5. Pre‐ and Post‐Challenge Stress Responses in Different Tissues

Bacterial infection triggered elevations in plasma cortisol and glucose, classical primary and secondary stress responses mobilizing energy via hepatic gluconeogenesis and glycogenolysis [83]. However, EE‐fed fish exhibited lower post‐challenge cortisol and glucose levels than controls, indicating an attenuated stress response. Herbal additives can lower glucose by strengthening antioxidant protection, supporting pancreatic insulin production [84], as previously observed in common carp [85].

Mitigating excessive cortisol and hyperglycemia is vital because prolonged stress suppresses immunocompetence and impairs growth [83]. Two potential mechanisms explain this stress reduction in EE‐fed fish: (1) direct in vitro antibacterial activity against *A. hydrophila* reduced systemic bacterial burden and inflammatory signaling and (2) potent antioxidant capacity limited oxidative tissue injury, thereby lowering the metabolic and energetic costs required for tissue repair. However, these hypothesis must be tested in the future for confirmation.

In the random forest model, plasma cortisol exhibited relatively low predictive importance for post‐challenge mortality. This suggests that cortisol reduction was a secondary consequence of improved redox balance and immune function rather than the primary driver of survival per se.

A comparative assessment of physiological parameters reveals that those exhibiting both interactive and main effects share a common biological signal. Across all categories, EE demonstrated a definitive protective effect against infection‐derived shock. Regarding EE concentrations, stress markers (glucose and cortisol) improved equally across all extract levels (1%, 2%, and 4%). In contrast, tissue‐specific defense mechanisms required varying concentrations: systemic fluid markers (such as TAC and MDA) reached saturation at the 1% EE, whereas the full activation of antioxidant enzymes and the immune system (such as GPx and Ig) required higher concentrations (with an optimum of 2%). This distinction highlights the difference between the rapid buffering capacity of systemic fluids and the higher threshold required to induce antioxidant enzymes and cellular immune responses.

## 5. Limitations

Several limitations warrant caution in interpreting these findings. First, the absence of alkamide quantification in the extract limits our understanding of the specific bioactive contributions to the observed outcomes. The lack of a sham‐injected control hinder the isolation of injection‐specific effects. Furthermore, sampling at a single post‐challenge time point introduces potential survivor bias, while the absence of pathogen re‐isolation or in vivo bacterial quantification renders the proposed antibacterial mechanisms speculative. Mechanistic interpretations are further limited by the lack of direct molecular evidence (e.g., gene expression analysis). Statistically, the exploratory correlation and random forest analyses should be viewed with caution due to the risk of overfitting in a small dataset (*n* = 12), and the tested dose range precludes a definitive determination of the optimal inclusion level. Finally, these controlled laboratory findings may not be directly translatable to commercial aquaculture settings characterized by higher environmental variability and distinct pathogen pressures.

## 6. Conclusion

This study demonstrates that dietary EE supplementation at 1%–2% optimizes mucosal and systemic immunity, antioxidant status, and disease resistance in common carp, even though it did not significantly enhance growth. EE improved feed efficiency, strengthened skin mucosal immunocompetence, and maintained intestinal redox homeostasis (elevated SOD and GPx and reduced MDA). Following *A. hydrophila* challenge, EE‐fed fish exhibited reduced mortality, attenuated endocrine stress (lower cortisol and glucose), and superior preservation of antioxidant defenses. Dose‐response analysis revealed quadratic patterns for growth and FCR, with 2% EE yielding the best feed efficiency, while mucosal immune parameters reached a plateau at 1% inclusion. Notably, random forest analysis highlighted mucosal immunity (Ig, bactericidal activity, and lysozyme) as the primary correlate of survival, though these results should be interpreted as hypothesis‐generating given the sample size. Future research should prioritize (1) refining the optimal dose through broader dose‐response trials, including lower (<1%) and intermediate concentrations; (2) assessing long‐term sustainability and stage‐specific efficacy; (3) investigating molecular mechanisms via gene expression (e.g., cytokines and tight junctions) and histopathology; (4) conducting digestibility studies to correlate improved FCR with nutrient absorption; and (5) quantifying in vivo pathogen load to elucidate antibacterial mechanisms. These findings position 1%–2% dietary EE (10–20 g/kg) as an effective strategy for enhancing carp health and stress resilience.

## Author Contributions

**Morteza Yousefi**: conceptualization, writing draft. **Hamed Abdollahpour**: formal analysis, visualization, writing draft. **Hossein Adineh**: project administration, investigation, methodology. **Sadaf Seyfollahi**: investigation, methodology. **Ali Taheri Mirghaed**: conceptualization, investigation. **Seyyed Morteza Hoseini**: conceptualization, formal analysis, writing draft, final editing.

## Funding

No fund was received for this study.

## Conflicts of Interest

The authors declare no conflicts of interest.

## Data Availability

The data of this article are available upon request from the corresponding authors.
